# Clusters of parental socioeconomic status in early childhood and inherited risk for cerebrovascular disease until mid-life—Northern Finland Birth Cohort 1966

**DOI:** 10.1177/17474930241282521

**Published:** 2024-09-30

**Authors:** Veronika Hyytiäinen, Leena Ala-Mursula, Petteri Oura, Markus Paananen, Ville Karhunen, Harri Rusanen, Mirjam I Geerlings, Jouko Miettunen, Ina Rissanen

**Affiliations:** 1 Research Unit of Population Health, University of Oulu, Oulu, Finland; 2 Medical Research Center Oulu, Oulu University Hospital, Oulu, Finland; 3 Research Unit of Health Sciences and Technology, University of Oulu, Oulu, Finland; 4 Department of Forensic Medicine, University of Helsinki, Helsinki, Finland; 5 Forensic Medicine Unit, Finnish Institute for Health and Welfare, Helsinki, Finland; 6 Research Unit of Mathematical Sciences, University of Oulu, Oulu, Finland; 7 Department of Neurology, Oulu University Hospital, Oulu, Finland; 8 Department of General Practice, Amsterdam UMC, Amsterdam, The Netherlands; 9 Amsterdam Public Health, Aging & Later life and Personalized Medicine, Amsterdam, The Netherlands; 10 Amsterdam Neuroscience; Neurodegeneration, and Mood, Anxiety, Psychosis, Stress, and Sleep, Amsterdam, The Netherlands; 11 Julius Center for Health Sciences and Primary Care, UMC Utrecht, Utrecht, The Netherlands

**Keywords:** Cohort studies, stroke, cerebrovascular disease, socioeconomic status, polygenic risk score, parent–offspring linkage

## Abstract

**Background and Aims:**

The incidence of cerebrovascular disease (CVD) is rising among young adults (< 55 years). The risk for CVD starts to form in early childhood and is comprised of genetic and environmental risk factors. The aim of this study is to investigate the relationship between early family socioeconomic status (SES), inherited risk, and CVD until midlife.

**Methods:**

In the Northern Finland Birth Cohort 1966 of 12,058 children, individuals were followed from gestational period up to 54 years. We used previously published early family SES clusters, based on latent class analysis of a wide set of prenatally collected variables. We investigated inherited risk with polygenic risk score (PRS) and parental CVDs during follow-up. The associations of the five distinct clusters, inherited risk, and consequent risk for various types of CVDs until middle age were analyzed with Cox regression. All analyses were conducted first in the whole sample and then stratified by sex as is recommended in cardiovascular studies.

**Results:**

During the follow-up of 586,943 person-years, 512 CVDs occurred. No clear association between SES clusters and CVD were found. Higher PRS associated with any CVD (hazard ratio (HR) per 1 SD increase: 1.15; 95% confidence interval (CI): 1.02–1.31), and ischemic CVD (HR: 1.21; 1.05–1.40). We found no combined associations of early family SES and inherited risk for CVD.

**Conclusions:**

Inherited risk was associated with the risk for CVD in mid-life in Finnish population. We found no clear connection with early family SES and CVD. Being born to a specific SES group did not increase the effect of inherited risk.

**Data access statement:**

NFBC1966 data are available from the University of Oulu, Infrastructure for Population Studies for researchers who meet the criteria for accessing confidential data. In the use of data, we follow the EU general data protection regulation (679/2016) and Finnish Data Protection Act. Permission to use the data can be applied for research purposes from https://www.oulu.fi/nfbc.

## Introduction

Cerebrovascular disease (CVD) is the second leading cause of death and the third leading cause of loss in productive years worldwide.^1^ The incidence of CVD is rising among young adults (< 55 years), which highlights the need to research underlying risk factors starting from early life.^2^ For all ages, non-modifiable risk factors, such as socioeconomic status (SES) one is born into and genetic risk, account for 10% of CVD risk.^3^ It has been suggested that the role of non-modifiable risk factors would be higher in young adult CVD.^4^

Despite low SES in adulthood being a known risk factor for CVD,^5^ the risk of being born to low SES is poorly understood. The critical period model of chronic diseases suggests that early-life disadvantages result in long-term, irreversible biological effects that manifest over time as CVD.^6,7^ Family SES is also known to influence one’s own adulthood SES.^8^

The offspring also inherit the genetic risk factors for CVD from their parents. Studies on CVD risk factors such as smoking^9^ and high body mass index^10^ have shown additive effects on risk when having both high genetic risk and low SES.

## Aims and hypothesis

We investigate the relationship between early family SES, inherited risk, and CVD until midlife in women and men in a large, unselected birth cohort. First, we analyzed these associations in the whole sample. Then all analyses were conducted stratified by sex since it is recommended in studies of cardiovascular diseases^11^ and according to previous literature showing that the impact SES has on CVD and other cardiovascular disease risk is different between sexes.^12^ We hypothesized that low early-life SES would associate with increased CVD risk and that persons who have high inherited risk and who are born to low family SES have especially high risk for young adulthood CVD. We also expected women to be more vulnerable to negative effects of lower SES than men.

## Methods

The Northern Finland Birth Cohort 1966 (NFBC1966) is a prospective, population-based, birth cohort containing data on 12,055 pregnant women and their 12,058 live-born children in the provinces of Oulu and Lapland with an expected date of birth in 1966,^13,14^ comprising 95.6% of all children born in the area.^14^

**Figure 1.**
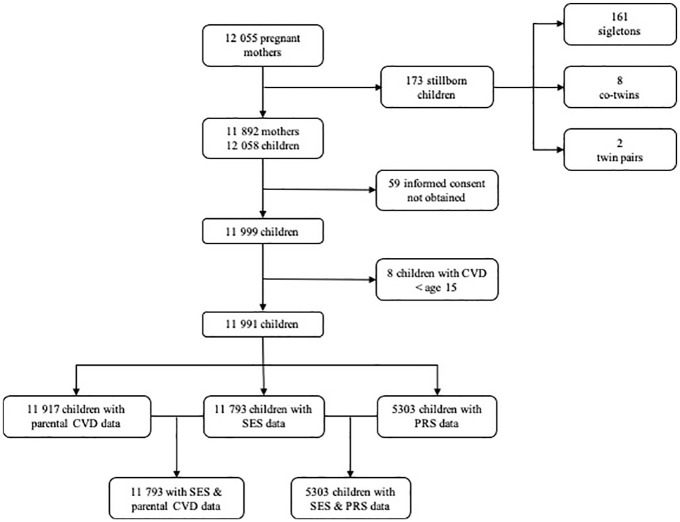
Population flow chart in Northern Finland Birth Cohort 1966. CVD: cerebrovascular disease; PRS: polygenic risk score; SES: socioeconomic status.

Excluded from the study were those children for whom informed consent to use their data was not available (*n* = 59), persons who had a CVD under the age of 15 years (*n* = 8), and persons who had moved abroad but whose moving date was unknown (*n* = 84). After this, data on SES were available for 11,793 individuals; polygenic risk score (PRS) for 5303 individuals; and parental CVD for 11,917 individuals (see Figure 1). For interaction analyses, both SES and PRS were available for 5303 and both SES and parental CVD for 11,793. The study population were followed from birth to their first CVD, death, moving abroad, or until 31 December 2020, whichever came first.

The study protocol follows the Declaration of Helsinki. Permission to gather register data was obtained from the Ministry of Social Affairs and Health, and the study was approved by the Northern Ostrobothnia Hospital District Ethical Committee 108/2017 (15 January 2018). Written informed consent was obtained from all the participants. This study was performed and reported in accordance with STROBE checklist for observational studies.^15^

### Cerebrovascular diseases

The information of CVDs, that is, strokes and transient ischemic attacks (TIAs), were obtained from the Care Register for Health Care and Causes of Death Register based on medical records. The linkage to questionnaire data with pseudonymization was fully complete for all CVD diagnoses. CVDs were classified by first primary diagnosis: subarachnoid hemorrhages (International Classification of Diseases, eighth revision (ICD-8): 430; ICD-9: 430; ICD-10: I60 and I69.0), intracerebral hemorrhages (ICD-8: 431; ICD-9: 431; ICD-10: I61 and I69.1), ischemic strokes (ICD-8: 432–434; ICD-9: 433–434; ICD-10: I63 and I69.3), TIA (ICD-8: 435; ICD-9: 435; ICD-10: G45), and other CVDs (ICD-8: 436–438; ICD-9: 436–437; ICD-10: I64–I68, I69.4, and I69.8). Other CVDs included, for example, non-ruptured cerebral aneurysms and central venous sinus thromboses. Stroke syndromes (ICD-9: 438; ICD-10: G46) were classified according to etiological sub-code (ICD-9: 430–437; ICD-10: I60–I67) or as other CVDs if subcodes were not present. Ischemic strokes and TIAs were defined as ischemic CVDs, and subarachnoid hemorrhages and intracerebral hemorrhages as hemorrhagic CVDs. For analyses of any CVD, ischemic CVDs, hemorrhagic CVDs, and other CVDs were combined. Traumatic subarachnoid hemorrhage or intracerebral hemorrhage, epidural hematoma, or subdural hematoma were not considered as CVDs. Subjects with two or more diagnoses were classified according to their principal diagnosis of the primary cerebrovascular event.

### Socioeconomic status

Previously published clusters based on a wide set of prenatally collected SES variables were used to describe the early family SES.^16^ The SES data were collected from questionnaires issued to the mothers during the 24th–28th weeks of pregnancy, from maternity cards, and from birth certificates of the offspring. SES variables included mother’s marital status, education, and occupation; father’s occupation; number of family members and those aged ⩽ 15 years; location of residence, room count, utilities; and family’s wealth. With latent class analysis (LCA), five SES clusters were found and they were named describing the distributions of the studied variables in each of them. For missing data, full information maximum likelihood method was used to compute parameter estimates based on all available data assuming data to be missing at random (0.0–10.1% per category). The clusters and their main characteristics are (according to Oura et al.^16^):

1. Highest status families (*n* = 1821, 15.4% of the population): Families characterized by high occupational status of the parents, regardless of the number of children. Located in cities or population centers. Large residences and above-average wealth.
2. Small families (*n* = 2598, 22.0%): Families with ⩽ 2 members and no individuals aged ⩽ 15 at the time of pregnancy. Living in a small residence in a city or population center.
3. Larger families (*n* = 1811, 15.4%): Families with ⩾ 5 members and ⩾ 3 individuals aged ⩽ 15, regardless of utilities.
4. Average wealth families (*n* = 2815, 23.9%): Medium-sized families living in medium-sized residences with average utilities and wealth.
5. Rural families (*n* = 2748, 23.3%): Relatively large families located in periphery, with farming as the most common occupation among parents.

The clusters were not considered to be entirely hierarchical. Highest status families were chosen to be the reference group. As a sensitivity analysis, we combined the four other clusters and analyzed their association to CVD compared to highest status families (results presented in the supplement). This can also be used for comparison with earlier studies on SES. The more detailed description of clusters and their composition with LCA is described elsewhere.^16^

### Genetic risk

We estimated inherited risk for CVD with PRS or as parental history of CVD. PRS is a weighted sum of genetic variants that associate with the risk of the phenotype of interest. The weights for the genetic variants were obtained from a genome-wide association study on CVD risk of 40,585 cases (any CVD) and 406,111 controls of European ancestry.^17^

The genotyping for NFBC1966 data has been previously described.^18^ The genomic variants were imputed against Haplotype Reference Consortium reference panel. We excluded variants with minor allele frequency < 0.05, or Hardy–Weinberg equilibrium *p*-value < 10^–6^, leaving 5,396,383 variants in the data.

PRSice software version 2.3.5 was used to calculate the PRS.^19^ The variants were clumped at *r*^2^ < 0.1 within 250 kb window, and candidate PRS was calculated using variants with different *p*-value cut-offs: 5 × 10^–8^, 5 × 10^–5^, and from 10^–3^ to 0.5 with increments of 0.001. The PRS with the highest *R*^2^ with the target phenotype, adjusted for sex and the first 20 genetic principal components, was selected for the subsequent association analyses. The PRS was standardized to mean of zero and unit standard deviation (SD). The *p*-value cut-off for the best PRS predictive performance was at 5 × 10^–5^, with Nagelkerke *R*^2^ = 0.3%.

Information of the parental CVDs was obtained from the Care Register for Health Care and Causes of Death Register based on medical records for which the follow-up commenced in 1967 and lasted until the end of the year 2020. The parental CVD diagnoses were collected and classified similar to the offspring’s diagnoses. We created two groups based on whether either of the parents had any kind of CVD or not.

### Statistical analyses

Characteristics of SES clusters were studied using one-way analysis of variance (ANOVA) and chi-square tests. Associations between early family SES, inherited CVD risk, and the offspring’s CVD outcomes were analyzed with Cox proportional hazards models. First, we performed independent analyses for associations of SES, PRS, or parental CVD and the offspring’s risk for CVD. Second, we tested multiplicative and additive interaction measures of SES × PRS, and SES × parental CVD, to investigate the combined associations of SES and inherited risk. Results are presented as hazard ratios (HRs) and relative excess risk due to interaction (RERI) and their 95% confidence intervals (CIs). The 95% CIs for the RERIs were calculted with delta method. We visually estimated that proportional hazards assumption was not violated. We performed main analyses both in the whole population and stratified by sex. We used externally and visually defined sex (women or men) at the time of birth without information of social gender. The models involving PRS were adjusted with the first five genetic ancestry principal components. We did not adjust the models for other factors, such as maternal smoking, health behavior, or own SES in adulthood since they were considered as effect mediators. We conducted Bonferroni correction for multiple testing when applicable.

As sensitivity analyses, we combined the four other clusters and analyzed their association to CVD compared to highest status families (see Supplemental material). Also, we analyzed the combined associations of SES and inherited risk by stratifying the population to low and high inherited risk according to PRS and parental CVD (see Supplemental material).

The data were accessed and analyzed using SPSS version 27 (IBM, Armonk, NY, USA). The RERIs were analyzed with R 4.3.1.

## Results

Characteristics of SES clusters are presented in Table 1. The total length of follow-up was 586,943 person-years. PRS was somewhat lower in the reference SES cluster of highest status families compared to other SES clusters, upon direct comparison (*p* = 0.07; one-way ANOVA). Instead, parental CVD risk was the highest within the highest status families in both sexes (*p* < 0.001; chi-square).

**Table 1.** Population characteristics and the number of people in socioeconomic clusters.

All (*N* = 11,793)	Highest status families	Small families	Larger families	Average wealth families	Rural families	*p*-value (chi-square/ANOVA)
	*n* (%)/mean (SD)	*n* (%)/mean (SD)	*n* (%)/mean (SD)	*n* (%)/mean (SD)	*n* (%)/mean (SD)	
Women (*n* = 5744) (48.7%)	874 (48.0)	1282 (49.3)	861 (47.5)	1364 (48.5)	1363 (49.6)	0.61
Mean PRS (*n* = 5303)	–0.09 (1.01)	0.03 (1.01)	0.01 (0.97)	0.00 (1.01)	0.02 (1.00)	0.07
Parental CVD	810 (44.5)	844 (32.5)	700 (38.7)	1025 (36.4)	1118 (40.7)	< 0.001
Mother’s CVD	475 (26.1)	429 (16.5)	471 (26.0)	577 (20.5)	713 (25.9)	< 0.001
Father’s CVD	452 (24.8)	507 (19.5)	327 (18.1)	572 (20.3)	547 (19.9)	< 0.001

ANOVA: analysis of variance; PRS: polygenic risk score, standardized to mean of zero and unit variance; CVD: cerebrovascular disease.

Of our study population, 512 (4.3%) had CVD during follow-up. Of them, 158 (30.9%) were ischemic CVDs, 196 (38.3%) TIAs, 61 (11.9%) subarachnoid hemorrhages, 39 (7.6%) intracerebral hemorrhages, and 58 (11.3%) other cerebrovascular events. The median incident age was 47.1 (SD 7.4) years for ischemic CVD, 48.8 (SD 5.3) years for TIA, 47.5 (SD 8.7) years for intracerebral hemorrhage, and 43.9 (SD 10.2) years for subarachnoid hemorrhage.

### SES, inherited risk, and CVD

We found no clear association between early family SES and any CVD, ischemic CVD, or hemorrhagic CVD in the total sample, in women or in men (see Table 2). This was also the case for sensitivity analyses where we compared the other clusters combined to the highest status families cluster (see Supplemental Table S1).

**Table 2.** Incidences and hazard ratios of CVD according to sex and SES clusters.

	Any CVD		Ischemic CVD		Hemorrhagic CVD	
	*N* (%) of CVDs	HR (95% CI)	*N* (%) of CVDs	HR (95% CI)	*N* (%) of CVDs	HR (95% CI)
All (*N* = 11,793)	*N* = 512		*N* = 354		*N* = 100	
Highest status families	72 (4.0)	Ref	49 (2.7)	Ref	15 (0.9)	Ref
Small families	118 (4.5)	1.21 (0.91–1.63)	82 (3.2)	1.24 (0.87–1.76)	23 (0.9)	1.14 (0.60–2.19)
Larger families	88 (4.9)	1.34 (0.98–1.83)	64 (3.6)	1.44 (0.99–2.08)	18 (1.0)	1.33 (0.67–2.63)
Average wealth families	117 (4.2)	1.13 (0.84–1.52)	83 (3.0)	1.18 (0.83–1.68)	21 (0.8)	0.98 (0.50–1.89)
Rural families	117 (4.3)	1.09 (0.81–1.46)	76 (2.8)	1.04 (0.73–1.49)	23 (0.9)	1.03 (0.54–1.97)
Women (*n* = 5744)	252 (4.4)		173 (3.1)		50 (0.9)	
Highest status families	31 (3.5)	Ref	19 (2.2)	Ref	9 (1.1)	Ref
Small families	62 (4.8)	1.44 (0.93–2.21)	43 (3.4)	1.62 (0.95–2.79)	11 (0.9)	0.89 (0.37–2.15)
Larger families	38 (4.4)	1.33 (0.83–2.13)	26 (3.1)	1.48 (0.82–2.67)	8 (1.0)	0.97 (0.38–2.52)
Average wealth families	57 (4.2)	1.24 (0.80–1.93)	41 (3.0)	1.46 (0.85–2.51)	10 (0.8)	0.76 (0.31–1.87)
Rural families	64 (4.7)	1.31 (0.85–2.01)	44 (3.3)	1.47 (0.86–2.51)	12 (0.9)	0.85 (0.36–2.02)
Men (*n* = 6049)	260 (4.3)		181 (3.0)		50 (0.9)	
Highest status families	41 (4.3)	Ref	30 (3.2)	Ref	6 (0.7)	Ref
Small families	56 (4.3)	1.04 (0.70–1.56)	39 (3.0)	0.99 (0.62–1.60)	12 (0.9)	1.52 (0.57–4.05)
Larger families	50 (5.3)	1.36 (0.90–2.05)	38 (4.1)	1.41 (0.88–2.28)	10 (1.1)	1.85 (0.67–5.09)
Average wealth families	60 (4.1)	1.05 (0.70–1.56)	42 (2.9)	1.00 (0.63–1.60)	11 (0.8)	1.30 (0.48–3.51)
Rural families	53 (3.8)	0.91 (0.61–1.37)	32 (2.3)	0.75 (0.46–1.24)	11 (0.8)	1.29 (0.48–3.48)

CVD: cerebrovascular disease; SES: socioeconomic status; HR: hazard ratio; CI: confidence interval; Ref: reference group.

Higher PRS was associated with increased risk for any CVD (HR: 1.15; 95% CI: 1.02–1.31) and for ischemic CVD (1.21; 1.05–1.40) in the total population. These estimates were somewhat higher in women, for any CVD (1.25; 1.06–1.49), ischemic CVD (1.27; 1.04–1.54), and hemorrhagic (1.77; 1.14–2.75) but no clear evidence for association was detected in men (see Table 3). Parental CVD was associated with ischemic CVD in men (1.39; 1.04–1.54). No clear association of parental CVD and SES was detected in the total population or in women. After Bonferroni correction for multiple testing, these results were diminished (results not shown).

**Table 3.** Hazard ratios of CVD according to sex, PRS, and parental CVD.

	Any CVD		Ischemic CVD		Hemorrhagic CVD	
	*N* (%) of CVDs	HR (95% CI)	*N* (%) of CVDs	HR (95% CI)	*N* (%) of CVDs	HR (95% CI)
All						
PRS (*N* = 5303)	260 (4.9)	1.15 (1.02–1.31)	192 (3.7)	1.21 (1.05–1.40)	40 (0.8)	1.28 (0.93–1.75)
Parental CVD (*N* = 11,917)	513 (4.3)	1.07 (0.89–1.27)	354 (3.0)	1.11 (0.90–1.37)	101 (0.8)	0.86 (0.58–1.29)
Women						
PRS (*n* = 2749)	138 (5.0)	1.25 (1.06–1.49)	105 (3.9)	1.27 (1.04–1.54)	21 (0.8)	1.77 (1.14–2.75)
Parental CVD (*n* = 5813)	252 (4.3)	0.94 (0.73–1.20)	173 (3.0)	0.86 (0.63–1.17)	50 (0.9)	1.15 (0.66–2.00)
Men						
PRS (*n* = 2554)	122 (4.8)	1.06 (0.88–1.27)	87 (3.5)	1.14 (0.92–1.41)	19 (0.8)	0.92 (0.59–1.44)
Parental CVD (*n* = 6104)	261 (4.3)	1.21 (0.95–1.54)	181 (3.0)	1.39 (1.04–1.87)	51 (0.8)	0.64 (0.35–1.16).

CVD: cerebrovascular disease; PRS: polygenic risk score; HR: hazard ratio; CI: confidence interval.

Table 4 shows the multiplicative interactions and RERIs of SES × PRS, and Table 5 the interaction measures of SES × parental CVD, in association to risk of CVD. We found no combined associations between early family SES and inherited risk in contrast to any CVD, ischemic CVD, or hemorrhagic CVD in the total sample, in women or in men. In total population, early family SES cluster of small families had a combined protective association with parental CVD to the hazard of any CVD (multiplicative interaction HR: 0.53; 0.29–0.98). After Bonferroni correction for multiple testing, this result was no longer significant (results not shown) and we consider this to be a probable chance result.

**Table 4.** Incidences and hazard ratios of CVD according to sex and interaction measures between SES and PRS.

	Any CVD			Ischemic CVD			Hemorrhagic CVD		
	*N* (%) of CVDs	MI HR (95% CI)	RERI (95% CI)	*N* (%) of CVDs	MI HR (95% CI)	RERI (95% CI)	*N* (%) of CVDs	MI HR (95% CI)	RERI (95% CI)
All (*N* = 5303)	260 (4.9)			192 (3.7)			40 (0.8)		
Highest status families × PRS	39 (4.7)	Ref	Ref	31 (3.8)	Ref	Ref	5 (0.6)	Ref	Ref
Small families × PRS	54 (4.8)	1.15 (0.76–1.75)	0.17 (–0.48; 0.82)	43 (3.9)	1.09 (0.68–1.74)	0.10 (–0.75; 0.96)	6 (0.6)	0.39 (0.41–4.66)	0.31 (–1.33; 1.95)
Larger families × PRS	45 (5.9)	0.87 (0.56–1.35)	–0.15 (–0.87; 0.58)	34 (4.5)	0.84 (0.51–1.38)	–0.19 (–1.13; 0.76)	8 (1.1)	0.71 (0.23–2.24)	–0.47 (–2.59; 1.65)
Average wealth families × PRS	61 (5.0)	0.88 (0.59–1.32)	–0.14 (–0.77; 0.50)	41 (3.4)	0.73 (0.46–1.17)	–0.35 (–1.15; 0.44)	11 (0.9)	1.97 (0.67–5.8)	1.22 (–1.42; 3.86)
Rural families × PRS	61 (4.5)	1.04 (0.69–1.56)	0.03 (–0.57; 0.63)	43 (3.2)	0.97 (0.60–1.54)	–0.09 (–0.86; 0.68)	10 (0.8)	0.86 (0.29–2.56)	–0.16 (–1.83; 1.51)
Women (*n* = 2749)	138 (5.0)			105 (3.9)			21 (0.8)		
Highest status families × PRS	18 (4.3)	Ref	Ref	13 (3.1)	Ref	Ref	4 (1.0)	Ref	Ref
Small families × PRS	34 (5.8)	1.30 (0.73–2.29)	0.45 (–0.66; 1.55)	27 (4.7)	1.62 (0.84–3.13)	0.82 (–0.56; 2.20)	4 (0.7)	0.91 (0.22–3.76)	–0.42 (–4.66; 3.82)
Larger families × PRS	20 (5.0)	0.94 (0.50–1.80)	–0.05 (–1.04; 0.93)	16 (4.0)	1.16 (0.55–2.43)	0.22 (–0.96; 1.40)	3 (0.8)	0.38 (0.08–1.82)	–1.30 (–5.68; 3.08)
Average wealth families × PRS	34 (5.4)	1.04 (0.59–1.84)	0.10 (–0.90; 1.09)	25 (4.0)	0.99 (0.51–1.91)	–0.01 (–1.03; 1.01)	5 (0.8)	1.94 (0.44–8.59)	0.33 (–4.10; 4.75)
Rural families × PRS	32 (4.5)	1.14 (0.64–2.03)	0.15 (–0.73; 1.03)	24 (3.4)	1.33 (0.68–2.60)	0.33 (–0.65; 1.31)	5 (0.7)	0.57 (0.15–2.20)	–0.93 (–5.07; 3.21)
Men (*n* = 2554)	122 (4.8)			87 (3.5)			19 (0.8)		
Highest status families × PRS	21 (5.1)	Ref	Ref	18 (4.4)	Ref	Ref	1 (0.3)	Ref	Ref
Small families × PRS	20 (3.7)	0.95 (0.51–1.78)	–0.10 (–0.91; 0.71)	16 (3.0)	0.67 (0.33–1.35)	–0.56 (–1.97; 0.84)	2 (0.4)	NA	NA
Larger families × PRS	25 (7.0)	0.83 (0.45–1.51)	–0.21 (–1.29; 0.86)	18 (5.1)	0.64 (0.32–1.28)	–0.57 (–2.25; 1.10)	5 (1.5)	NA	NA
Average wealth families × PRS	27 (4.5)	0.72 (0.41–1.29)	–0.32 (–1.17; 0.53)	16 (2.7)	0.55 (0.27–1.11)	–0.71 (–2.11; 0.69)	6 (1.0)	NA	NA
Rural families × PRS	29 (4.5)	0.96 (0.54–1.70)	–0.08 (–0.91; 0.76)	19 (3.0)	0.72 (0.37–1.40)	–0.51 (–1.91; 0.89)	5 (0.8)	NA	NA

CVD: cerebrovascular disease; SES: socioeconomic status; PRS: polygenic risk score; MI: multiplicative interaction; HR: hazard ratio; CI: confidence interval; RERI: relative excess risk due to interaction; Ref: reference group; NA: not applicable.

**Table 5.** Incidences and hazard ratios of CVD according to sex and interaction measures between SES and parental CVD.

	Any CVD			Ischemic CVD			Hemorrhagic CVD		
	*N* (%) of CVDs	MI HR (95% CI)	RERI (95% CI)	*N* (%) of CVDs	MI HR (95% CI)	RERI (95% CI)	*N* (%) of CVDs	MI HR (95% CI)	RERI (95% CI)
All (*N* = 5303)	512 (4.3)			354 (3.0)			100 (0.9)		
Highest status families × parental CVD	72 (4.0)	Ref	Ref	49 (2.7)	Ref	Ref	15 (0.9)	Ref	Ref
Small families × parental CVD	118 (4.5)	0.53 (0.29–0.98)	–0.84 (–1.84; 0.16)	82 (3.2)	0.60 (0.20–1.24)	–0.66 (–1.84; 0.51)	23 (0.9)	0.28 (0.07–1.19)	–1.74 (–4.53; 1.04)
Larger families × parental CVD	88 (4.9)	0.72 (0.39–1.35)	–0.35 (–1.35; 0.66)	64 (3.6)	0.67 (0.32–1.42)	–0.46 (–1.73; 0.80)	18 (1.0)	0.61 (0.15–2.44)	–0.63 (–3.11; 1.84)
Average wealth families × parental CVD	117 (4.2)	0.67 (0.37–1.22)	–0.50 (–1.40; 0.40)	83 (3.0)	0.60 (0.29–1.23)	–0.66 (–1.81; 0.48)	21 (0.8)	0.52 (0.13–2.02)	–0.90 (–3.16; 1.37)
Rural families × parental CVD	117 (4.3)	0.60 (0.33–1.08)	–0.67 (–1.59; 0.25)	76 (2.8)	0.72 (0.35–1.49)	–0.41 (–1.46; 0.64)	23 (0.9)	0.33 (0.08–1.28)	–1.52 (–4.12; 1.09)
Women (*n* = 5744)	252 (4.4)			173 (3.1)			50 (0.9)		
Highest status families × parental CVD	31 (3.5)	Ref	Ref	19 (2.3)	Ref	Ref	9 (1.1)	Ref	Ref
Small families × parental CVD	62 (4.8)	0.45 (0.18–1.10)	–1.19 (–2.91; 0.53)	43 (3.4)	0.61 (0.20–1.84)	–0.70 (–2.52; 1.12)	11 (0.9)	0.26 (0.04–1.77)	–2.1 (–6.49; 2.29)
Larger families × parental CVD	38 (4.4)	0.68 (0.26–1.76)	–0.45 (–2.06; 1.15)	26 (3.1)	0.65 (0.20–2.16)	–0.56 (–2.45; 1.34)	8 (1.0)	0.95 (0.13–6.96)	–0.08 (–3.48; 3.31)
Average wealth families × parental CVD	57 (4.2)	0.48 (0.20–1.19)	–1.02 (–2.62; 0.59)	41 (3.0)	0.46 (0.15–1.42)	–1.10 (–3.02; 0.81)	10 (0.8)	0.61 (0.10–3.92)	–0.95 (–4.37; 2.47)
Rural families × parental CVD	64 (4.7)	0.46 (0.19–1.11)	–1.11 (–2.74 0.52)	44 (3.3)	0.66 (0.22–1.96)	–0.53 (–2.2; 1.14)	12 (0.9)	0.18 (0.03–1.18)	–2.55 (–7.30; 2.20)
Men (*n* = 6049)	260 (4.3)			181 (3.0)			50 (0.9)		
Highest status families × parental CVD	41 (4.3)	Ref	Ref	30 (3.2)	Ref	Ref	6 (0.7)	Ref	Ref
Small families × parental CVD	56 (4.3)	0.63 (0.27–1.45)	–0.58 (–1.77; 0.62)	39 (3.0)	0.63 (0.24–1.69)	–0.61 (–2.15; 0.92)	12 (0.9)	0.34 (0.04–3.06)	–1.43 (–4.96; 2.09)
Larger families × parental CVD	50 (5.3)	0.76 (0.33–1.74)	–0.27 (–1.56; 1.01)	38 (4.1)	0.68 (0.26–1.80)	–0.40 (–2.09; 1.30)	10 (1.1)	0.49 (0.06–3.98)	–1.21 (–4.95; 2.54)
Average wealth families × parental CVD	60 (4.1)	0.90 (0.40–1.99)	–0.11 (–1.17; 0.96)	42 (2.9)	0.79 (0.31–2.05)	–0.30 (–1.71; 1.11)	11 (0.8)	0.49 (0.06–3.95)	–0.89 (–3.89; 2.12)
Rural families × parental CVD	53 (3.8)	0.78 (0.34–1.76)	–0.33 (–1.4; 0.74)	32 (2.3)	0.90 (0.33–2.47)	–0.28 (–1.62; 1.05)	11 (0.8)	0.68 (0.09–5.12)	–0.48 (–3.2; 2.24)

CVD: cerebrovascular disease; SES: socioeconomic status; MI: multiplicative interaction; HR: hazard ratio; CI: confidence interval; RERI: relative excess risk due to interaction; Ref: reference group.

Associations between SES and any CVD, ischemic CVD, and hemorrhagic CVD stratified by low, intermediate, and high PRS are shown in Supplemental Table S2, and stratified by parental CVD in Supplemental Table S3. No clear associations between SES clusters and CVD risk were found in analyses stratified by PRS. In analyses stratified by parental CVD, in the group of no parental CVD, the SES clusters of small families (HR: 1.63; 1.08–2.46) and larger families (HR: 1.60; 1.02–2.52) were associated with increased risk for any CVD and the cluster of larger families with increased risk for ischemic CVD (HR: 1.77; 1.04–3.04). No robust associations were found when a person had parental history of CVD. After Bonferroni correction, these results were no longer significant.

## Discussion

In this large, population-based birth cohort of nearly 12,000 individuals, we examined clusters of early family SES and inherited risk factors associated with CVD in mid-life in Finnish population. Early family SES was not robustly associated with CVD risk. Instead, inherited risk was associated with CVD in mid-life. Furthermore, there was no clear evidence of an interactive association of any SES group on inherited risk in either sex.

To date, most studies that investigated the relation between early family SES and CVD risk in adulthood have focused on a few socioeconomic variables. To our knowledge, this is the first study where early family SES is described broadly with clusters. The measuring of SES is challenging, since it is context-dependent and multidimensional. Some of its dimensions are more sensitive to real-life changes in socioeconomic hierarchies than others. Indeed, the overall SES includes both vertically hierarchical and horizontally different dimensions, yet nevertheless depicts an individual’s position in the socioeconomic hierarchy of the society. As sensitivity analysis, we combined the four other clusters and compared them to the highest status families cluster to aid with comparison to previous studies on SES.

We found no clear association between early family SES and adult CVD risk. Mechanisms behind this finding are mostly still unknown. However, disparities between the SES clusters are rather narrow in Northern Finland, which might explain the uniformity in cerebrovascular health.

Expectedly, inherited risk, described by genetic risk and parental CVD, associated with later CVD in our study population. This is in line with previous studies showing that both genetic risk^20,21^ and parental CVD^22–24^ are risk factors for CVD, especially among young CVD patients.^4^ Despite PRS being a common and efficient way of illustrating the genetic risk for a given phenotype on an individual level, it captures only a fraction of CVD heritability, which is the case in many diseases with a polygenic etiology.^20^ Acknowledging that family histories and PRS do not provide identical information,^25,26^ we used parental CVD as another proxy for heritability. It should also be noted that a more recent genome-wide association study (GWAS) with 7,588,359 single-nucleotide polymorphisms (SNPs) was published after the beginning of this project.^27^ However, the majority of new SNPs are from a non-European origin and therefore would not have remarkably influenced our results. In future studies, we recommend using the GWAS from the study by Mishra et al.

We found no clear multiplicative nor additive associations of SES and inherited risk for CVD. This may be explained by SES not showing a clear independent connection on CVD risk. The combined association of parental CVD and small family SES cluster was protective from offspring’s any CVD. However, after Bonferroni correction, this finding was no longer significant and we consider this to be a probable chance result. In stratified analyses, we found increased risk for CVD in offspring of small and larger families with no parental CVD. No associations were found when parents had CVD. Future studies should further investigate the possible role of parental CVD as an effect modifier between the association of SES and risk for CVD. It should be noted that in our study the follow-up for parental CVDs commenced in 1967, meanwhile the questionnaire with information of SES was issued in 1965. Therefore, causality cannot be seen between parental CVD and their possibly low SES. In addition, there are no records of parental CVDs before the time of birth or pregnancy. We found no robust multiplicative nor additive associations of SES and PRS for CVD.

Previous studies have shown that SES has different impacts on CVD risk between women and men.^28^ Furthermore, the interpretation of socioeconomic measures might differ between sexes.^29^ Therefore, we investigated associations between SES, inherited risk, and risk for CVD separately in women and men. We discovered that PRS seems to be a risk factor for CVD in women, but not in men. On contrast, parental CVD seems to be a risk factor for ischemic CVD in men. Noteworthy, we had information only on the biological sex and could not investigate the effects of gender.

### Strengths and limitations

Our main strength is the use of a large, unselected, population-based birth cohort with almost 12,000 participants and 585,000 person-years of follow-up. Data collection started from the second trimester of the antenatal period and the cohort members have been followed up ever since. We were also able to perform analyses separately for women and men. Information on CVD diagnoses was complete for the entire cohort from nationwide registers. The accuracy and validity of these registers and CVD diagnoses is estimated to be fairly good, with around 85% sensitivity and positive predictive value for any type of first CVD event in Finnish population.^30,31^ We had comprehensive data on the cohort member’s early family environment and were able to model early family SES with clusters. We had access to PRS and parental CVD data.

Our main limitation was the small number of cases for the CVD subtypes. CVD in early adulthood is rare, and even though we had a large-scale cohort with comprehensive follow-up data, only 512 CVDs were recorded. Concerning early family SES measures, we were limited to accuracy of data collected in the 1960s. In addition, the genetic data were obtained from less than half of the cohort members. A larger sample was achieved with parental CVDs. However, we had no information of parental CVDs before the year 1967.

This study also had the limitations of an epidemiological cohort study setting. We performed analyses stratified by sex, but otherwise the population was assumed to be homogeneous or the possible differences to be a consequence of being born to a specific SES cluster or having different inherited risk. We did not adjust the models with other factors, such as pregnancy complications, adulthood SES, or lifestyle and health factors, since we considered them as effect mediators. Investigating SES is always dependent of the era, area, and cultural aspects of the society and population. Furthermore, we conducted multiple analyses and after Bonferroni correction our findings were slightly diminished.

In this study, we have aimed to emphasize the perspective of lifelong health and the role of innate and environmental risk factors to CVD risk. Our unique SES cluster model is an example of a multidimensional and more realistic approach to SES, and it should be applied in other sociocultural contexts. In conclusion, we found no clear connection with early family SES and CVD in mid-life contrary to our hypothesis. Our results show that inherited risk was associated with the risk for CVD. Being born to a specific SES group did not increase the effect of inherited risk. Further studies are needed to investigate inherited risk and other environmental factors combined with later-life SES and their associations to CVD risk and its risk factors.

## Supplemental Material

sj-docx-1-wso-10.1177_17474930241282521 – Supplemental material for Clusters of parental socioeconomic status in early childhood and inherited risk for cerebrovascular disease until mid-life—Northern Finland Birth Cohort 1966 Supplemental material, sj-docx-1-wso-10.1177_17474930241282521 for Clusters of parental socioeconomic status in early childhood and inherited risk for cerebrovascular disease until mid-life—Northern Finland Birth Cohort 1966 by Veronika Hyytiäinen, Leena Ala-Mursula, Petteri Oura, Markus Paananen, Ville Karhunen, Harri Rusanen, Mirjam I Geerlings, Jouko Miettunen and Ina Rissanen in International Journal of Stroke
